# Acetylsalicylic acid inhibits the growth of melanoma tumors via SOX2-dependent-PAF-R-independent signaling pathway

**DOI:** 10.18632/oncotarget.18326

**Published:** 2017-06-01

**Authors:** Anita Thyagarajan, Jeremiah Saylae, Ravi P. Sahu

**Affiliations:** ^1^ Department of Pharmacology and Toxicology, Boonshoft School of Medicine at Wright State University, Dayton, OH, USA

**Keywords:** acetylsalicylic acid, melanoma, platelet-activating factor-receptor, prostaglandin F2 alpha, SOX2

## Abstract

Acquired resistance to standard therapies remains a serious challenge, requiring novel therapeutic approaches that incorporate potential factors involved in tumor resistance. As cancers including melanoma express inflammatory cyclooxygenases generating prostaglandins implicated in tumor growth, we investigated mechanism of anti-inflammatory drug, acetylsalicylic acid (ASA) which has been shown to inhibit various tumor types, however, its effects against highly aggressive melanoma model are unclear. Given our reports that an activation of platelet-activating factor-receptor (PAF-R) augments the growth and impede efficacies of therapeutic agents in experimental melanoma, we also sought to determine if PAF-R mediates anti-melanoma activity of ASA. The current studies using stably PAF-R-positive (B16-PAFR) and negative (B16-MSCV) murine melanoma cells and PAF-R-expressing and deficient mice, demonstrate that ASA inhibits the *in-vitro* and *in-vivo* growth of highly aggressive B16F10 melanoma via bypassing tumoral or stromal PAF-R signaling. Similar ASA-induced effects *in-vitro* were seen in human melanoma and nasopharyngeal carcinoma cells positive or negative in PAF-R. Mechanistically, the ASA-induced decrease in cell survival and increase in apoptosis were significantly blocked by prostaglandin F2 alpha (PGF2α) agonists. Importantly, PCR array and qRT-PCR analysis of B16-tumors revealed significant downregulation of sry-related high-mobility-box-2 (SOX2) oncogene by ASA treatment. Interestingly, modulation of SOX2 expression by PGF2α agonists and upregulation by fibroblast growth factor 1 (FGF-1) rescued melanoma cells from ASA-induced decreased survival and increased apoptosis. Moreover, PGF2α-receptor antagonist, AL8810 mimics ASA-induced decreased melanoma cells survival which was significantly blocked by PGF2α and FGF-1. These findings indicate that ASA inhibits the growth of aggressive melanoma via SOX2-dependent-PAF-R-indepedent pathway.

## INTRODUCTION

Malignant melanoma is the most aggressive type of skin cancer that contributes about 80% of skin-cancer-related deaths leading to over 9000 annual mortality in the United States [1]. Melanomas are often resistant to standard therapies including the new targeted therapies, indicating the need of novel approaches for its intervention [2–6]. In contrast, immunotherapy strategies have shown the most promise as the immune responses are critical for the eradication of melanoma [7–8]. Importantly, most standard therapies for cancers including melanoma also act as potent pro-oxidative stressors by which they eradicate tumor cells as one of the potent mechanisms [9–13].

Our previous studies have shown that an activation of platelet-activating factor-receptor (PAF-R), a seven transmembrane G-protein coupled receptor mediates pro-oxidative stressors-induced acute pro-inflammatory and delayed systemic immunosuppressive responses [12–23]. The PAF-R is expressed on various immune and non-immune cells including keratinocytes and tumor cells [12–23]. Importantly, PAF-R activation by pro-oxidative stressors including the therapeutic agents, augment the growth and impede cancer therapy efficacy in experimental murine melanoma models in a process blocked by cyclooxygenase type 2 (COX-2) inhibitors [16–19]. Cyclooxygenases (COX) enzyme catalyzes the conversion of arachidonic acid to prostaglandins (PGs) [24–26].

Most importantly, malignancies including melanoma express COX-2 and, inhibition of COX-2 or its generated PGs have been shown to exert promising anti-tumor effects against melanoma [24–27]. Prostaglandin E2 (PGE2) and prostaglandin F2 alpha (PGF2α) are the major PGs involved in mediating several behaviors of cancer cells including the cell proliferation and invasion [26, 45]. Thus, there has been considerable rationale for the use of anti-cancer drug(s) targeting COX-2 or PGs for cancer chemoprevention [28–30].

Notably, aspirin, an acetylsalicylic acid (ASA) has classically been used as an anti-inflammatory drug due to its ability to inhibit COX enzymes resulting in decreased synthesis of PGs, and thus, possesses chemopreventive activities against a variety of cancers [30–36]. While some studies have demonstrated ASA-effects in syngeneic murine B16F0 or B16F1 melanoma models [37–40], its role and mechanism in a highly aggressive and syngeneic B16F10 murine melanoma model that resembles the aggressive and advanced melanoma in humans are unclear. Of significance, in clinical studies, where administration of ASA has been shown to reduce the risk of human cancers including gastric and colon cancer, there have been mixed results regarding the use of ASA and melanoma risk [41–43]. Thus, an investigation of ASA mechanism of action in a highly aggressive melanoma model is necessary to further evaluate its efficacy, identify novel target(s) and define the rationale for its use in combination therapy.

Among various factors involved in inducing tumor resistance or anti-apoptotic responses to standard therapies against cancers, sry-related high-mobility box -2 (SOX-2) gene has emerged as an important oncogene which affects tumor cell behavior including proliferation, apoptosis and resistance to standard therapies [44–47]. While SOX2 expression has been identified in cancer models [44–49], there is only limited information of SOX2 in melanoma. Moreover, the role of ASA in modulating SOX2 expression in melanoma model has not been studied.

As ASA targets both COX-dependent and independent pathways [50–51], the current studies sought to determine the role and mechanism of ASA in B16F10 melanoma model. The present studies describe a novel mechanism by which ASA suppresses the *in-vitro* and *in-vivo* growth B16F10 melanoma tumors via SOX2-dependent-PAF-R-independent pathway.

## RESULTS

### Aspirin inhibits the survival of murine melanoma cells via inducing apoptosis

**Figure 1:**
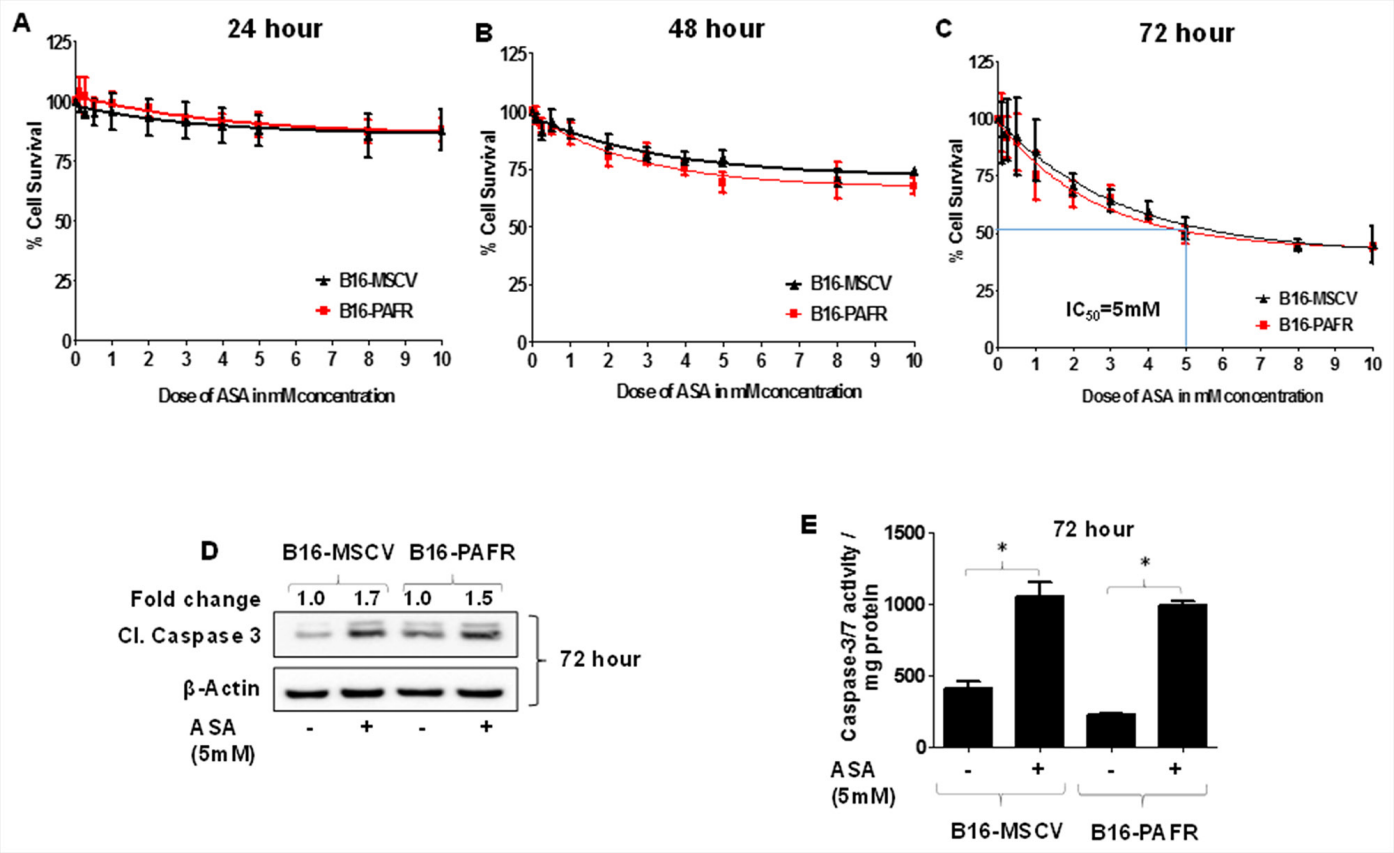
Effect of aspirin treatment on the growth of B16F10 melanoma cells expressing or deficient in PAF-R **(A-C)** B16F10 cells stably expressing PAF-R (B16-PAFR) or deficient, vector control (B16-MSCV) cells were treated with various doses of ASA (0.1, 0.25, 0.5, 1.0, 2.0, 3.0, 4.0, 5.0, 8.0 and 10 mM) and cultured for 24, 48 and 72 hour. Control cells were treated with 0.1% DMSO only. At these time points, cells were fixed and cell survival was assessed via staining with sulforhodamine B (SRB) as described. **(D-E)** B16-MSCV and B16-PAFR cells were treated with 0.1% DMSO or 5 mM ASA and cultured for 72 hour followed by the detection of cleaved caspase-3 for apoptosis induction normalized to β-actin for control by western blotting (the fold change in cleaved caspase 3 between DMSO and ASA treated cells is shown for quantification) or luminescence-based caspase-3/7 activity normalized to 0.5 mg of total protein by plate reader as described. Data are the mean ± SD and expressed as % cell survival or caspase 3/7 activity/mg protein over the doses of ASA from at least three separate experiments. Statistical significant difference (p<0.05) was noted in caspase 3/7 activity between vehicle and ASA-treated B16-MSCV and B16-PAFR cells (Figure 1E).

**Figure 2:**
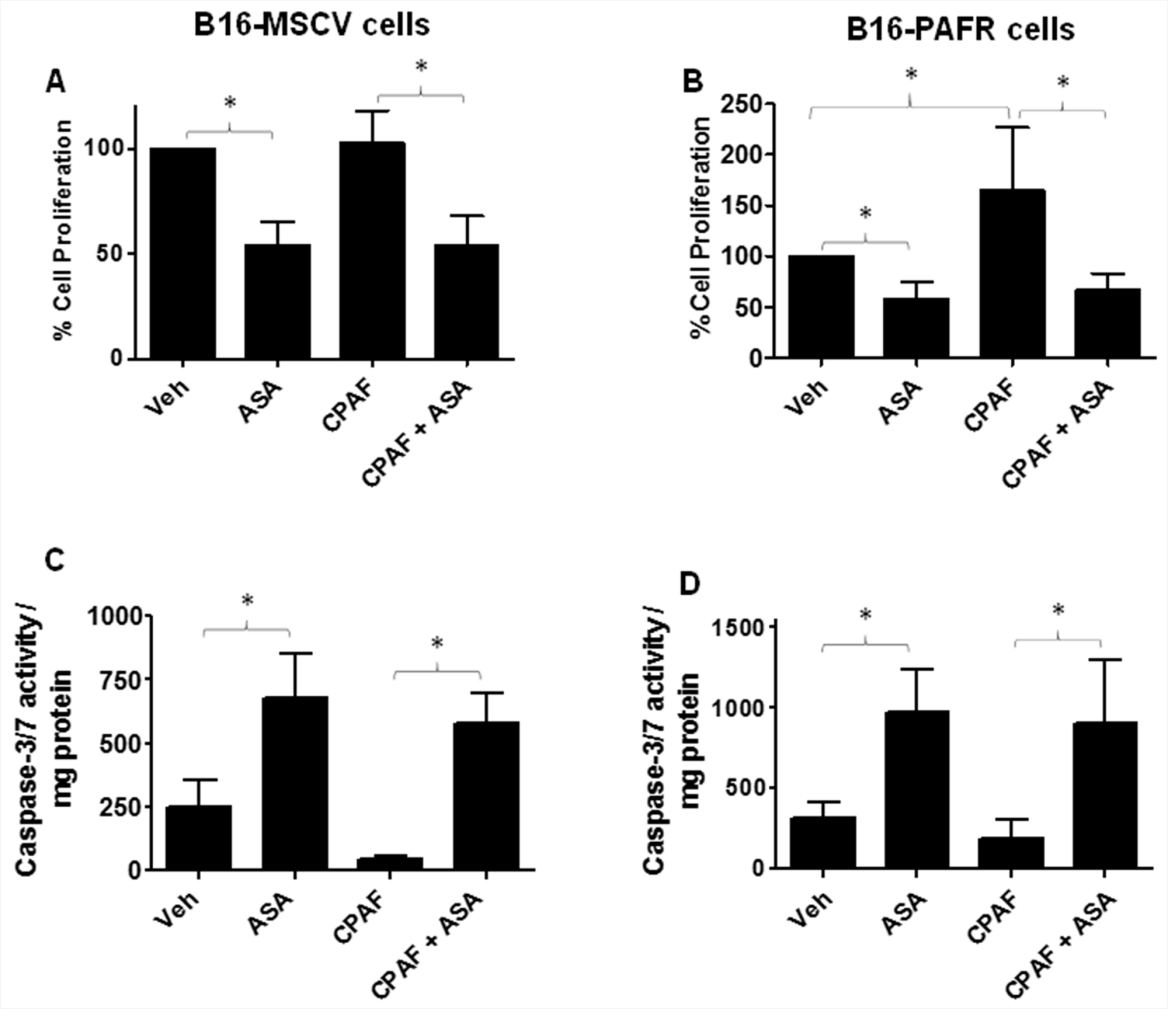
Effect of functional PAF-R activation on ASA-induced effects **(A-D)** B16-MSCV and B16-PAFR cells were treated with or without 10 nM PAF-R agonist, CPAF and/or 5 mM ASA. Control cells received 0.1% DMSO only. After 72 hour, cell proliferation was measured by trypan blue exclusion assay and apoptosis by luminescence-based caspase-3/7 activity normalized to 0.5 mg protein as described. Data are the mean ± SD and expressed as % cell proliferation or caspase 3/7 activity/mg protein in various groups from at least three separate experiments.

Given our studies demonstrating the role of PAF-R signaling in melanoma tumor growth [16–20], we first tested the involvement of PAF-R pathway in ASA-induced effects. To that end, we utilized stable PAF-R-positive (B16-PAFR) and negative (B16-MSCV, for control) cells generated via retroviral-mediated transduction of parent B16F10 melanoma cells. The generation and characterization of these cells have been described by us [17]. As shown in Figure 1A-1C, ASA treatment (0.1 to 10 mM) suppressed the survival of these cells at similar rates in a dose and time dependent manner with an IC_50_ of 5mM at 72 hours as measured via a quantitative sulforhodamine (SRB) assay [52]. Our next studies used the IC_50_ dose of ASA at 72 hour time point and demonstrated that ASA-mediated inhibition of these melanoma cell survival was due to the induction of apoptosis as measured by immunoblotting for cleaved caspase 3, luminescence and fluorescence-based caspase-3/7 activity (Figure 1D-1E and Supplementary Figure 1). Our next functional studies investigated if PAF-R activation by known PAF-R agonist, CPAF could rescue ASA-induced decreased growth and increased apoptosis of melanoma cells. We observed that while CPAF-alone treatment increased the proliferation of B16-PAFR cells, it did not rescue the cells from ASA-induced reduced proliferation and induction of apoptosis (Figure 2A-2D). Similarly, ASA treatments inhibited the survival of human PAF-R-negative SK5MEL melanoma and PAF-R-positive KBP and negative KBM nasopharyngeal carcinoma cells (Supplementary Figure 2A-2F) generated via retroviral-mediated transduction as reported by us [18–19, 52]. These findings indicate that ASA-induced effects bypass cellular-PAF-R signaling in melanoma cells and these effects are not specific to murine melanoma.

### ASA suppresses the *in-vivo* growth of melanoma tumors

**Figure 3:**
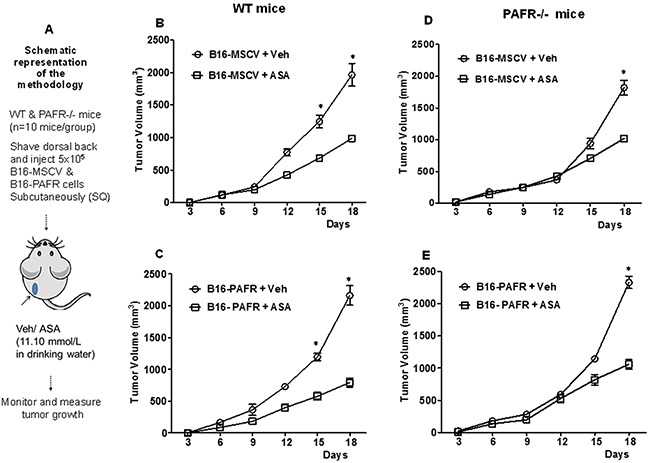
Effect of ASA on *in-vivo* growth of melanoma tumors in WT and PAFR−/− mice **(A)** Groups of PAF-R expressing C57BL/6-WT or deficient (PAFR−/−) mice (n=10 mice per group) were implanted with 5×10^5^ B16-MSCV or B16-PAFR tumor cells into the shaved dorsal hind flanks. These mice were treated with 0.2% DMSO (as vehicle control) or 11.10 mmol/L ASA dissolved in 0.2% DMSO in drinking water and monitored for tumor development. **(B-E)** Tumor growth was assessed by measuring major and minor circumferences with digital caliper at every 3 days and tumor volume was calculated. Data are the mean ± SE and expressed as tumor volume (mm^3^) over the period of time (days). Statistical significant differences (p<0.05) were noted between vehicle and ASA-treated WT and PAFR−/− mice at days 15 and/or 18.

We have shown that an activation of stromal (host)-PAF-R mediates augmentation of melanoma tumor growth [17]. Our next studies determined effects of ASA on *in-vivo* melanoma tumor growth with the focus of investigating the role of host-PAF-R. To that end, we first performed pilot studies to determine the optimal dose of ASA that inhibits melanoma tumor growth. Our pilot studies in WT mice (n=5/group) demonstrate that supplementation of ASA (2.8, 5.6 and 11.1 mmol/L in 0.2% DMSO) in drinking water inhibits B16-MSCV tumor growth compared to vehicle (0.2% DMSO) in a dose-dependent manner without exerting deleterious effects (Supplementary Figure 3A-3B). The greater tumor suppressive effects were seen with 11.1 mmol/L dose of ASA. We next evaluated effects of 11.1 mmol/L dose of ASA on the growth of B16-MSCV and B16-PAFR tumors in WT mice (n=10 mice per groups) as outlined in Figure 3A. We observed that ASA-treatment significantly suppressed the growth of both B16-MSCV and B16-PAFR tumors at similar rates indicating that ASA-induced effects bypass cellular and host-PAF-R (Figure 3B-3C). To further confirm the involvement of host-PAF-R signaling, we performed studies in PAF-R deficient (PAFR−/−) mice (10 mice/group) and observed similar growth inhibition of B16-MSCV and B16-PAFR tumors by ASA (Figure 3D-3E). These studies indicate that ASA-induced suppression of *in-vivo* melanoma tumor growth bypasses cellular and stromal PAFR signaling.

### PGF2α but not PGE2 protected melanoma cells from ASA-induced decreased survival

**Figure 4:**
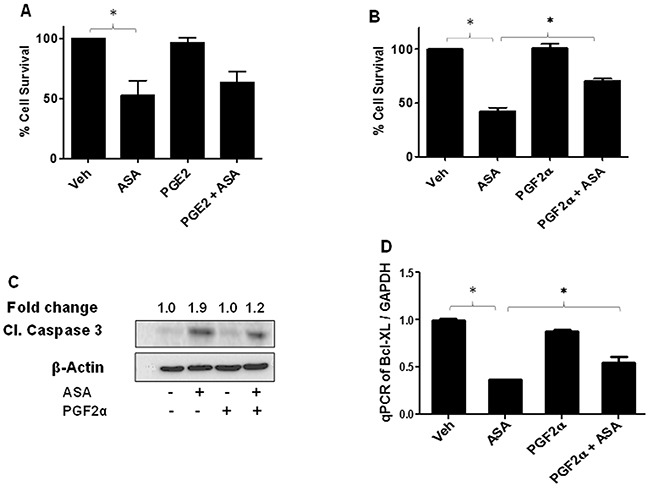
Effect of PGE2 and PGF2α on ASA-induced effects on cell survival and apoptosis induction B16-MSCV cells were pretreated with PGE2 or PGF2α at 1.0 μg/ml dose and left for 24 hour followed by treatment with or without ASA (5 mM) and cultured for 72 hour. **(A-B)** % cell survival was measured by SRB assay, **(C)** cleaved caspase 3 by western blotting as described. The fold change in cleaved caspase 3 between ASA ± PGF2α treated cells is shown for quantification. **(D)** Total RNA was extracted from these treatments and cDNA samples were analyzed for Bcl-XL by qRT-PCR and normalized to GAPDH. Data are the mean ± SD and expressed as % cell survival, aspase 3/7 activity/mg protein or Bcl-XL/GAPDH in various groups from at least three separate experiments.

Among various PGs, PGE2 and PGF2α are the major PGs involved in mediating several behaviors of cancer cells including cell proliferation and invasion [26, 50]. Our next studies with an objectives of determining the mechanism of ASA-induced decreased growth of melanoma cells, evaluated the roles of PGE2 and PGF2α. Our further *in-vitro* studies used B16-MSCV cells with ASA at IC_50_ dose of 5mM at 72 hour. To that end, we performed initial studies to test various working doses of PGE2 and PGF2α which by itself do not affect the cell viability (data not shown) and chosen 1.0 μg/ml dose. Next, B16-MSCV cells were cultured with PGE2 and PGF2α at 1.0 μg/ml concentration and cultured for 24 hour followed by treatment with or without ASA at 5mM dose and cultured for 72 hour. We observed that PGF2α but not PGE2 significantly protected B16-MSCV cells from ASA-induced reduced survival (Figure 4A-4B). We next tested if PGF2α could rescue the cells from ASA-induced apoptosis. As shown in Figure 4C, ASA-induced increase cleavage of caspase-3 was significantly blocked by PGF2α agonist, indicating its potential role in ASA-induced apoptosis. Our studies also demonstrate an inhibition of anti-apoptotic protein Bcl-XL by ASA which was significantly rescued by PGF2α indicating its role in protecting melanoma cells from ASA-induced apoptosis (Figure 4D).

### SOX2 mediates ASA-induced decreased growth of melanoma tumors *in-vivo*

**Figure 5:**
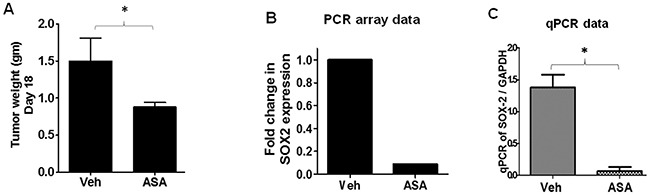
PCR array and qRT-PCR analysis of B16-tumors from vehicle and ASA-treated WT mice B16-MSCV tumors from WT mice (n=10) treated with or without ASA (11.10 mmol/L) were used to extract total RNA. **(A)** Tumor weight at day 18 between vehicle and ASA-treated groups. **(B)** RNA samples from vehicle vs ASA-treated tumors were pooled separately and synthesized cDNA was blotted into separate PCR array plates. The fold change in SOX2 gene expression is shown. **(C)** The RNA samples of each tumor (n=10) were analyzed separately for the SOX2 gene by qRT-PCR and normalized to GAPDH to further confirm the PCR array data. The data are represented as mean ± SD and statistical significant difference (p<0.05) was observed in SOX2 gene between vehicle and ASA-treated group.

As COX-independent mechanisms have also been proposed for ASA-induced effects [50–51], our next approach was to identify the target(s) that mediate ASA-induced reduced growth of melanoma tumors. To that end, we performed PCR array analysis using B16-MSCV tumors isolated from vehicle and ASA-treated WT mice (n=10 mice/group) (Figure 5A) as described in the method section. While the expression of most of the genes were unaltered, we observed 10 fold downregulation of sry-related high-mobility-box-2 (SOX2) oncogene by ASA treatment compared to control (Figure 5B). We confirmed this finding by performing separate qRT-PCR studies for SOX2 gene in individual B16-MSCV tumors from vehicle and ASA-treated WT mice and observed similar findings (Figure 5C). These studies indicate that SOX2 mediates ASA-induced decreased growth of melanoma tumors and thus, further studies were focused on SOX2.

### PGF2α modulates SOX2 in mediating ASA-induced effects

**Figure 6:**
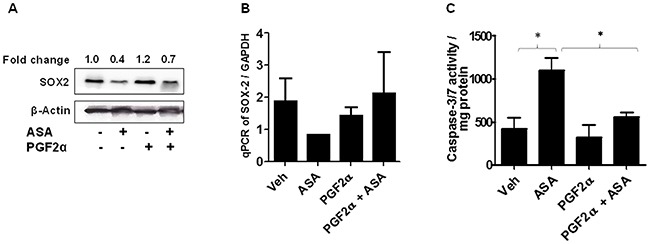
Effect of PGF2α on SOX2 expression and ASA-induced apoptosis **(A-B)** B16-MSCV cells were pretreated with PGF2α (1.0 μg/ml) and left for 24 hour followed by treatment with or without ASA (5 mM). After 72 hour, cells were used to extract protein lysates and total RNA. The protein lysates were blotted with anti-SOX2 antibody and β-actin for equal loading control. The fold change in SOX2 between ASA ± PGF2α treated cells is shown for quantification. The expression of SOX2 gene was analyzed from cDNA by qRT-PCR and normalized to GAPDH. **(C)** The protein lysates were extracted and 0.5 mg protein from different groups were incubated with caspase -3/7 buffer and activity was assessed via measuring luminescence by plate reader. The data are from three separate experiments and expressed as **(A)** SOX2 & β-actin, **(B)** mean ± SD of SOX2/GAPDH and **(C)** caspase-3/7 activity/mg protein over various groups. Statistical significant difference (p<0.05) was observed between vehicle and ASA or ASA and PGF2α + ASA-treated groups.

Our next studies determined if PGF2α can modulate SOX2 in mediating ASA-induced effects. To that end, B16-MSCV cells were pretreated PGF2α agonist (1.0 μg/ml) and left for 24 hour followed by treatment with 0.1% DMSO or 5mM ASA. After 72 hours, the analysis of SOX2 gene was performed by qRT-PCR and western blotting. As shown in Figure 6A-6B, treatment with ASA decreased the expression and protein levels of SOX2, further confirming the *in-vivo* PCR array data with B16-MSCV tumors (Figure 5B-5C). Importantly, PGF2α treatment attenuated ASA-induced reduction in SOX2 gene expression (Figure 6A-6B) and apoptosis induction (Figure 6C).

### SOX-2 upregulation blocks ASA-induced effects

**Figure 7:**
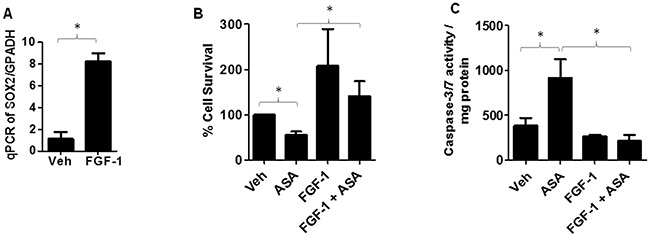
Effect of FGF-1 on SOX2 expression and ASA-induced effects **(A)** B16-MSCV cells were pre-treated with FGF-1 (10 ng/ml) and left for 24 hour followed by extraction of RNA and, synthesized cDNA was analyzed for SOX2 gene expression by qRT-PCR and data normalized to GAPDH. **(B)** B16-MSCV cells were pretreated with FGF-1 (10 ng/ml) and left for 24 hour followed by treatment with or without ASA (5 mM) and cultured for 72 hour. The cell survival was assessed by SRB assay. **(C)** The protein lysates were extracted and 0.5 mg protein from different groups were incubated with caspase -3/7 buffer and activity was assessed via measuring luminescence by plate reader. The data are mean ± SD of three separate experiments and expressed as SOX2/GAPDH **(A)**, % cell survival **(B)** and caspase-3/7 activity/mg protein **(C)** over various groups. Statistical significant difference (p<0.05) was observed between vehicle and FGF-1 or ASA and FGF-1 + ASA-treated groups.

We next investigated if SOX2 upregulation can rescue melanoma cells from ASA-induced inhibition of cell survival and induction of apoptosis. Notably, fibroblast growth factor-1 (FGF-1) treatment has been shown to upregulate SOX2 expression in murine primary calvarial osteoblast cells in a time dependent manner with substantial increase was noted at 24 hour [53]. We first tested if the same dosing regimen (10ng/ml) of FGF-1 at 24 hour could induce SOX2 in melanoma cells? To that end, B16-MSCV cells were treated with or without FGF-1 and after 24 hours, total RNA was extracted and cDNA samples were analyzed for SOX2 expression by qRT-PCR and normalized to GAPDH. As shown in Figure 7A, we observed significant upregulation in SOX2 gene expression. In next studies, B16-MSCV cells were pretreated with FGF-1 (10ng/ml) and after 24 hour treated with 0.1% DMSO or 5mM ASA and cultured for 72 hour followed by assessments of cell survival and apoptosis by SRB and caspase-3/7 activity assays. Our studies demonstrate that FGF-1 treatment that upregulates SOX2, significantly blocked ASA-induced reduction in cell survival and induction of apoptosis (Figure 7B-7C). These findings indicate that SOX2 mediates ASA-induced decreased growth of murine melanoma cells.

### SOX-2 upregulation blocks PGF2α mimetic-induced effects

**Figure 8:**
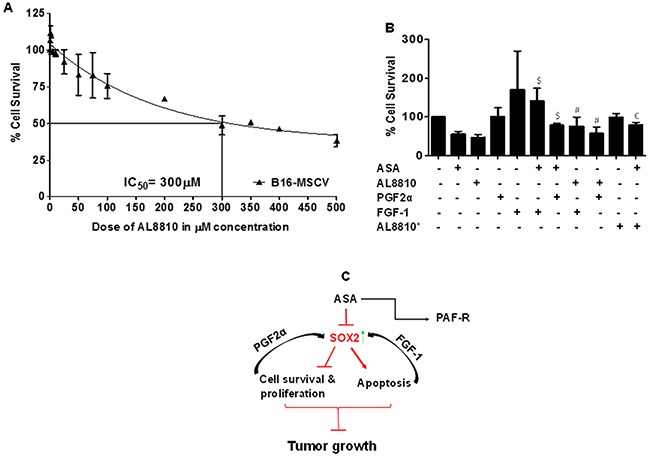
Effect of PGF2α and FGF-1 on PGF2α-FP receptor antagonist or ASA-induced effects on the growth of melanoma cells **(A)** B16-MSCV cells were treated with or without various doses of PGF2α-FP receptor antagonist, AL8810 (0.5, 1, 2.5, 5, 10, 25, 50, 75, 100, 200, 300, 350, 400 and 500 μM) and cultured for 72 hour. **(B)** B16-MSCV cells were pretreated with PGF2α (1.0 μg/ml) or FGF-1 (10 ng/ml) and left for 24 hour followed by treatment with or without AL8810 (0.5 μM and 300 μM) and ASA (5 mM). In both these experiments, control cells received 0.1% DMSO only and after 72 hour, % cell survival was assessed by SRB assay as described. The data are mean ± SD of three independent experiments and expressed as % cell survival over various doses of AL8810 or various groups. The statistical significant differences (P<0.05) were noted between ASA and PGF2α + ASA and FGF-1 + ASA groups (denoted by $), and AL8810* (0.5 μM) and AL8810* + ASA groups (denoted by $) and between AL8810 (300 μM) and PGF2α + AL8810 and FGF-1 + AL8810 groups (denoted by #). **(C)** The schematic representation of working model is shown.

As ASA-induced effects were blocked by modulation of SOX2 by PGF2α and FGF-1 in melanoma cells (Figures 6-7), we next investigated if PGF2α-FP receptor antagonist, AL8810 [54–55] can mimic ASA effects and if SOX-2 upregulation can abrogate its effect? To that end, our first studies evaluated effects of AL8810 on the survival of B16-MSCV cells. For this, melanoma cells were treated with 0.1% DMSO or various doses of AL8810 (0.5μM to 500μM) and after 72 hour, cell survival was analyzed by SRB assay. As shown in Figure 8A, AL8810 treatment reduces the survival of B16-MSCV cells in a dose dependent manner with an IC50 of 300μM. Our next studies determined if PGF2α and FGF-1 that modulate SOX2 expression, can rescue melanoma cells from AL8810-induced inhibition of survival, and compared the responses with ASA. For this, B16-MSCV cells were pretreated with PGF2α and FGF-1 and left for 24 hour followed by the treatments with 0.1% DMSO, AL8810 (300μM) or ASA (5mM). After 72 hours, cell survival was assessed by SRB assay. As shown in Figure 8B, both PGF2α and FGF-1 significantly attenuated AL8810-induced reduced survival of melanoma cells similar to as seen with ASA-treatment. In addition, pretreatment with AL8810 at lower dose (0.5 μM, denoted by *) that does not affect the cell viability, significantly attenuated ASA-induced inhibition of cell survival (Figure 8B) indicating that blockade of FP receptor abrogates ASA-induced effects in B16-MSCV cells. These findings suggest that SOX2 signaling pathway mediates ASA-induced decreased growth of melanoma growth (Figure 8C).

## DISCUSSION

Acetylsalicylic acid (ASA), the common non-steroidal anti-inflammatory (NSAID) household drug has been used for decades as an anti-inflammatory, analgesic and anti-pyretic agent [30, 50]. In addition, ASA effects as an antithrombotic agent have been established against stroke and cardiovascular diseases [56–57]. Importantly, while clinical studies have demonstrated that administration of ASA reduces the risk of human cancers including gastric and colon cancer, mixed responses have been reported regarding ASA in reducing the risk of melanoma [41–43]. Thus, an evaluation and identification of novel target(s) of ASA in highly aggressive syngeneic melanoma model will result in new therapeutic strategy for melanoma chemoprevention.

The present studies tested the role and mechanisms of ASA in highly aggressive and syngeneic B16F10 melanoma model. Notably, where B16F0 and B16F1 murine melanoma cells are non-aggressive, B16F10 cells are the highly aggressive murine melanoma model which resembles the malignant stage of advanced melanoma in humans. Importantly, published studies have used either B16F0 or B16F1 melanoma cells in determining ASA-induced effects on *in-vitro* and/or *in-vivo* melanoma growth [37–40]. Our studies using murine B16F10 melanoma cells demonstrated that ASA inhibits the *in-vitro* and *in-vivo* growth of melanoma tumors via bypassing the PAF-R pathway. These studies are consistent with our published report demonstrating that augmentation of murine EL4 lymphoma tumor growth bypasses the PAF-R signaling [16]. As induction of apoptosis is an important phenomenon in mediating the chemopreventive activities of anti-tumor agents [11–12, 50, 52], our studies demonstrate that ASA-treatment induces apoptosis to inhibit the survival of melanoma cells. These findings are consistent with other studies demonstrating that ASA induced apoptosis to inhibit the growth of cancer cells [32–33, 35].

Prostaglandin-endoperoxide synthase or COX is an enzyme with multiple isoforms (COX-1 and COX-2) that catalyze the conversion of arachidonic acid to prostaglandins (PGs), one of the major pathways implicated in mediating inflammatory responses and/or affecting tumor growth [25–26, 50]. This inhibition of COX enzymes decreases the catalytic production of PGs and several studies have shown that COX-2 inhibition by ASA suppresses cancer growth [30, 32]. Among various PGs, PGE2 and PGF2α synthesis play crucial roles, particularly relevant to several cancer cell specific processes including proliferation, invasion and resistance to apoptosis [25, 29–30, 41]. Our studies demonstrate that pretreatment of PGF2α but not PGE2 significantly protected melanoma cells from ASA-induced decreased cell survival, Bcl-XL and increased apoptosis. Although significant but an incomplete rescue of ASA-induced effects on cell survival, Bcl-XL and apoptosis by PGF2α may suggest the involvement of other factor(s) in mediating ASA-induced effects which remains the goal of future studies. These findings are consistent with the studies of Tsai et al demonstrating that ASA-treatment decreased PGF2α production in B16F0 cells which reduced its migration and invasion via inhibiting matrix metalloproteinase (MMP)-2 activity [38].

Notably, COX-dependent and independent pathways have been proposed for ASA-induced effects in various tumor types including B16F0 and B16F1 melanoma models [30–40, 43, 50]. To identify the target(s) and mechanism of ASA involved in inhibiting the *in-vitro* and *in-vivo* growth of B16F10 melanoma, we took advantage of the PCR array analysis. Our studies demonstrate 10 fold downregulation of SOX2 gene by ASA treatment. Further qRT-PCR analysis of B16-MSCV tumors confirmed the PCR array data analysis for the expression of SOX2 gene.

SOX2 gene encodes for a transcription factor which belong to SOX gene family and functions as an activator or suppressor of the gene transcription [44]. Earlier studies have shown that SOX2 plays crucial role in several processes including stem cell maintenance and cellular reprograming and its alteration induces developmental maladies such as anophthalmia-esophageal-genital (AEG) syndrome [44, 53]. In regard to cancer, recent studies have shown that SOX2 is amplified in various cancer types that affect cancer cell physiology including proliferation, apoptosis and resistance to standard therapies [44–47]. While the role of SOX2 has been identified in various cancer models including melanoma [44–49], it is not studied whether ASA can modulate SOX2 expression in melanoma model. Our studies demonstrate that ASA treatment inhibits SOX2 gene expression and modulation of SOX2 by PGF2α attenuates ASA-induced inhibition of survival and induction of apoptosis in murine B16F10 melanoma cells. Importantly, SOX2 upregulation by FGF-1 promoted the survival of melanoma cells and significantly blocked ASA-induced reduced cell survival and increased apoptosis. These findings are in agreement with studies demonstrating that SOX2 promotes tumorigenesis and increases the anti-apoptotic property of cancer cells and its downregulation inhibits cancer growth [47–49]. Furthermore, our studies show that SOX-2 upregulation blocks FP receptor antagonist effects which mimics ASA-induced effects in inhibiting the survival of melanoma cells.

In summary, the present studies describe a novel mechanism by which ASA suppresses the *in-vitro* and *in-vivo* growth of highly aggressive B16F10 melanoma tumors via SOX2-dependent-PAF-R independent signaling pathway. Importantly, as SOX2 play crucial roles in tumor-resistance or anti-apoptotic responses to standard therapies, our studies indicate that anti-SOX2 targeting agent(s) could further be tested for melanoma chemoprevention.

## MATERIALS AND METHODS

### Materials and reagents

All chemicals were obtained from Sigma-Aldrich (St. Louis, MO) unless indicated otherwise. The Qiagen RNA extraction kit and RT^2^ Profiler^TM^ PCR array were purchased from Qiagen Sciences (Germantown, MD). The cDNA kit and SYBR green qPCR reagent were purchased from Life technologies (Carlsbad, CA). The PAF-R, SOX2 and GAPDH primers were obtained from SABiosciences (Valencia, CA). Caspase-3/7 activity assay kit was purchased from Promega Corporation (Madison, WI). Prostaglandin E2 (PGE2) and F2α (PGF2α) were purchased from Cayman Chemicals Co. (Ann Arbor, MI). Antibodies against cleaved caspase-3, SOX2, β-actin were purchased from Cell Signaling Technology (Danvers, MA).

### Cells

Murine parent PAF-R negative B16F10 melanoma cells obtained from ATCC (Manassas, VA, USA) used to generate stably PAF-R-expressing (B16-PAFR) and deficient (B16-MSCV) cells as described by us [17–18]. Similarly, PAF-R-positive and deficient KBP and KBM cells generated from PAF-R-negative human nasopharyngeal carcinoma KB cells were used in these studies [14–15, 17–19]. In addition, PAF-R-deficient human melanoma SK5MEL cells obtained from ATCC were used. These cells were maintained in DMEM media supplemented with 10% fetal bovine serum and 100 *μ*g/ml mixture of penicillin and streptomycin as described by us [14–15, 17–19, 52] and tested during the course of the study for their authenticity and were used at 12-32 passage numbers.

### Mice

PAF-R expressing C57BL/6 wildtype (WT) and PAF-R knockout mice (*Ptafr*−/−) mice (originally obtained from Dr. Satoshi Ishii, The University of Tokyo [58] on C57BL/6 background. These mice strains (50% males and females) at the age of 8-12 weeks were used in these studies. All mice were housed under specific pathogen-free conditions. All procedures were approved by the Institutional Animal Care and Use Committee of Wright State University.

### Cell survival assay

The B16-MSCV and B16-PAFR cells were seeded into 96-well plates (2×10^3^ cells/well) and cultured overnight followed by treatment with 0.1% DMSO or various concentrations of ASA (1-10 mM) in serum-free media, as indicated in respective figures and figure legends. The cell survival was assessed at 24 hour, 48 hour and 72 hour by sulforhodamine-B (SRB) assay, as described by us previously [52]. In separate experiments, B16-MSCV and B16-PAFR cells were cultured with or without PGE2 or PGF2α (1.0 μg/ml) for 24 hour followed by treatment with or without 5mM ASA and after 72 hour, cell survival was assessed. In addition, B16-MSCV cells were treated with 0.1% DMSO or various doses of AL8810 (0.5 μM to 500 μM) in serum-free media and after 72 hour cell survival was measured. Next, B16-MSCV cells were pretreated with PGF2α (1.0 μg/ml) or FGF-1 (10 ng/ml) and left for 24 hour followed by treatments with 0.1% DMSO or AL8810 (0.5 μM & 300 μM) or ASA (5 mM) in serum-free media and 72 hour later cell survival was analyzed by SRB assay [52].

### Cell proliferation assay

The B16-MSCV and B16-PAFR cells were plated in 24-well plates (2×10^4^ cells/well) and treated with 10 nM 1-hexadecyl-2-N-methylcarbamoy l-3-glycerophosphocholine (CPAF) and/or 5mM ASA and incubated for 72 hour. The control cells received 0.1% DMSO. After 72 hour, cells were trypsinized, washed and resuspended in media. The cell proliferation was assessed by standard trypan blue exclusion method using a Countess automated cell counter (Life technologies, Carlsbad, CA) [52].

### Apoptosis assay

The B16-MSCV or B16-PAF-R cells (1×10^5^ cells/well) were seeded into 6-well plates with or without PGF2α (1.0 μg/ml) and after 24 hour treated with 0.1% DMSO or 5 mM ASA in serum-free media. After 72 hour, cells were homogenized in hypotonic extraction buffer and 0.5 mg of total protein was incubated with caspase-3/7 glo reagent (Promega, Madison WI) as described by us [52, 59]. The caspase 3/7 activity was measured by Synergy H1 Luminescence microplate reader (BioTek, Winvooski, VT) at our Proteome Analysis Laboratory (PAL) core facilities. In addition, we confirmed ASA-induced apoptosis induction by detecting cleaved caspase-3 by western blotting and/or used fluorescence-based caspase-3/7 activity assay as markers of apoptosis using Incucyte instrument (Essen Bioscience) according to the manual's protocol. In separate experiments, B16-MSCV cells were pretreated with FGF-1 (10 ng/ml) and cultured for 24 hour followed by treatment with 0.1% DMSO or 5 mM ASA. After 72 hour, caspase-3/7 activity was assessed [52, 59].

### Western blot analysis

B16-PAF-R and/or B16-MSCV cells (0.75×10^6^) were seeded in 100mm disc with or without PGF2α (1.0 μg/ml) and cultured for 24 hour followed by treatment with 0.1% DMSO (vehicle) or 5mM ASA in serum free media and left for 72 hours. Whole-cell extracts were prepared and cell lysates containing 40-60 μg proteins were subjected to SDS-PAGE and blotted onto PVDF membrane for western blot analysis as described by us previously [59] for the analysis of cleaved caspase-3 and SOX2 and normalized by β-actin. The quantitation of immunoblots was done using Image lab 5.1 software from Biorad Chemidoc MP imaging system (Hercules, CA) at our Proteome Analysis Laboratory (PAL) core facilities.

### Quantitative real-time PCR (qPCR)

The mRNA expression of Bcl-XL and SOX2 was analyzed in reverse transcribed cDNA from DNA-digested total RNA extracted from B16-MSCV cells treated with 0.1% DMSO or 5 mM ASA using qPCR and the data were normalized with GAPDH as described by us [17, 59–60]. Similarly, B16-MSCV tumors (n=10 mice/group) harvested from vehicle vs ASA-treated (11.1 mmol/L) WT mice were analyzed for SOX2 mRNA expression. In separate experiment, B16-MSCV cells were treated with 0.1% DMSO or FGF-1 (10 ng/ml) and after 24 hour, total RNA was analyzed for SOX2 expression. The fluorescence was detected using a Step One plus Real-time PCR machine (Applied Biosystems, Foster City, CA). The quantification of each PCR product was performed by 2^−ΔΔCt^ method [17, 59–60].

### *In-Vivo* tumor growth studies

To determine effects of tumor versus stromal PAF-R expression in ASA-mediated modulation of experimental melanoma tumor growth, we first performed pilot studies with n=5 syngeneic WT mice and following 0.5 × 10^6^ B16-MSCV melanoma cells implantation, these mice were treated with varying doses of ASA (2.8, 5.6 and 11.1 mmol/L dissolved in drinking water with 0.2% DMSO) to determine the dose of ASA that inhibits melanoma tumor growth. Mice were monitored for tumor development and mice weights were recorded. Finally, we choose 11.1 mmol/L (dissolved in water with 0.2% DMSO). Next, 0.5 × 10^6^ murine B16-PAFR and B16-MSCV cells were implanted subcutaneously (SQ) into the shaved right hind flanks of WT and PAFR−/− mice (day 0) followed by treatment with or without ASA during the entire period of the experiment. Control mice received 0.2% DMSO in water as vehicle. Tumor growth (major and minor circumferences) in these studies was monitored and measured every 3 days with digital calipers (Mitutoyo Corp., Aurora, IL), and tumor volume was calculated (major circumference × minor circumference^2^/2). Additionally, mice and tumor weights were recorded by digital balance.

### PCR array analysis

To determine the ASA target(s), B16-MSCV tumors from vehicle and ASA-treated WT mice (n=10 mice/group) were harvested for PCR array analysis. Briefly, total RNA was extracted and 1μg of total RNA from each tumor sample was pooled separately from each group. Complementary DNA (cDNA) was synthesized using cDNA synthesis kit and blotted separately into RT^2^ Profiler^TM^ PCR array plates (PAMM-176Zc-2 for mouse cancer stem cells) and qPCR and analysis were performed as per manual's protocol and software.

### Statistical analysis

Statistical analysis was assessed by Graph Pad Prism 5.0 software. All *in-vitro* experiments were repeated independently at least three times and at least five mice/group were used in murine experiments. Data were analyzed by Student's t-test or one-way ANOVA with post hoc Bonferroni's or Newman-Keuls multiple comparison tests. Statistical significance was set as a p value <0.05.

## SUPPLEMENTARY FIGURES



## Abbreviations

ASA: acetylsalicylic acid
PAF: platelet-activating factor
PAF-R: PAF-receptor
CPAF: 1-hexadecyl-2-N-methylcarbamoyl glycerophosphocholine
PGE2: prostaglandin E2
PGF2α: prostaglandin F2 alpha
SOX2: sry-related high-mobility box -2
COX: cyclooxygenase
FGF-1: fibroblast growth factor 1
PGF2α-FP: PGF2α receptor
