# Supplementary material for: Acetylsalicylic acid inhibits the growth of melanoma tumors via SOX2-dependent-PAF-R-independent signaling pathway
Source: Oncotarget. 2017 Jun 1;8(30):49959–72. doi: 10.18632/oncotarget.18326 (PMC5564820; doi:10.18632/oncotarget.18326)
Supplement: Supplementary file 1 [file oncotarget-08-49959-s001.pdf]

Acetylsalicylic acid inhibits the growth of melanoma tumors via SOX2-dependent-PAF-R-independent signaling pathway

SUPPLEMENTARY MATERIALS

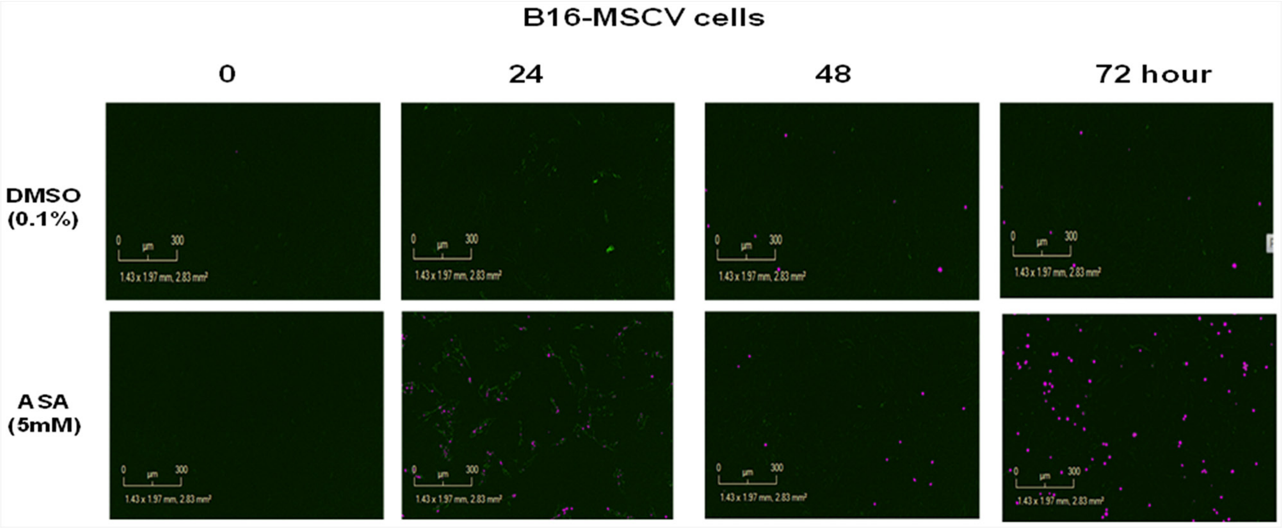

Supplementary Figure 1: Effect of ASA on caspase-3/7 apoptosis in B16-MSCV cells. B16-MSCV cells were seeded with caspase-3/7 reagent containing DEVD peptide, DEVD recognition motif and DNA binding dye) and cultured for overnight. After that the cells were treated with or without ASA (5mM) and left for 24, 48 and 72 hour. Control cells received 0.1% DMSO only. At these times points, the cells undergoing apoptosis were imaged for fluorescence detection within the Incucyte plate reader. The activated caspase-3/7 cleaves caspase-3/7 reagent at the DEVD recognition motif, releasing a DNA binding dye that fluorescently labels the nuclear DNA of apoptotic cells.

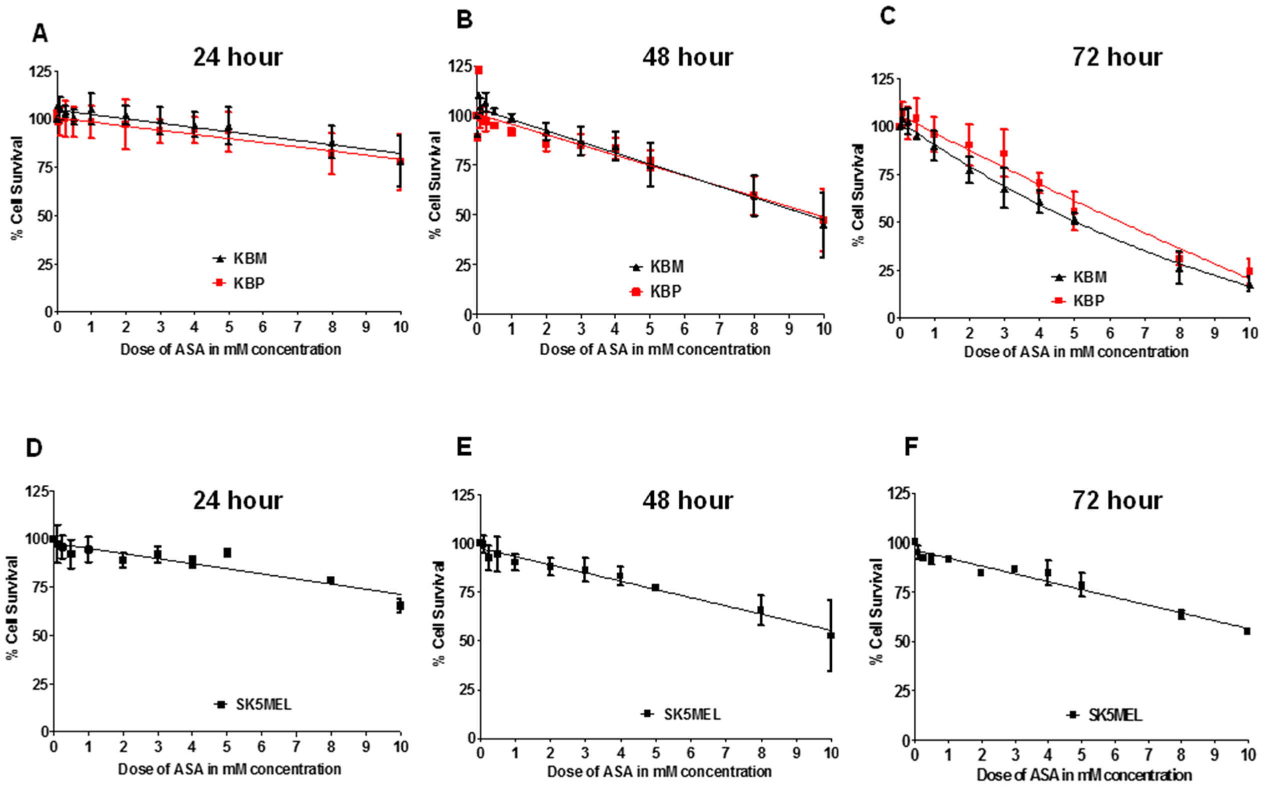

Supplementary Figure 2: Effect of ASA on the survival of human cancer cells expressing or deficient in PAF-R. **(A-C)** Human nasopharyngeal carcinoma cells stably expressing PAF-R (KBP) or deficient (KBM) cells were treated with or without various doses of ASA and left for 24, 48 and 72 hour. The cell survival was assessed by SRB assay as described. **(D-F)** Similarly, the effects of ASA on the cell survival was assessed in human PAF-R deficient, SK5MEL cells. The data are mean ± SD of three independent experiments and expressed as % cell survival over various doses of ASA.

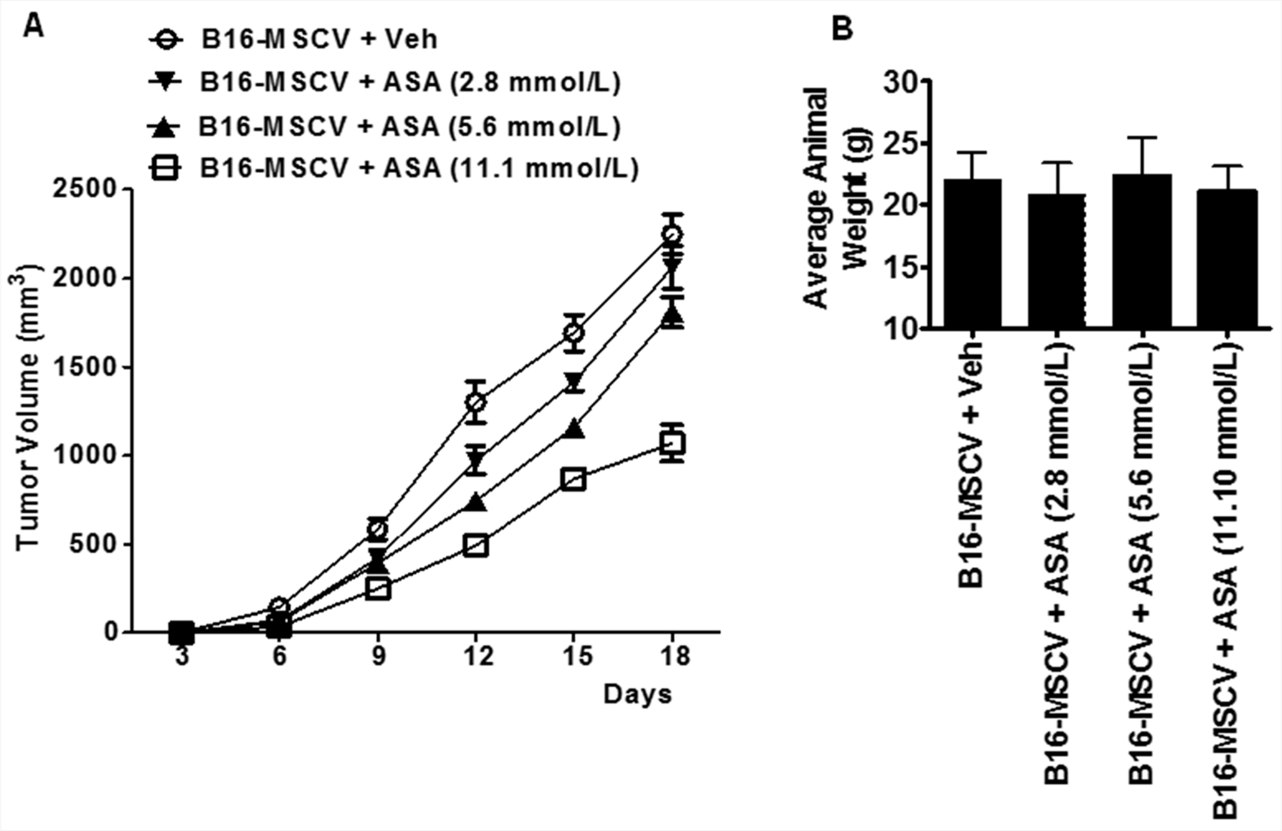

Supplementary Figure 3: Effects of various doses of ASA on the growth of B16-MSCV tumors in WT mice. **(A-B)** Groups of C57BL/6-WT mice (n=5) were implanted with 5x10^5 B16-MSCV cells into the shaved dorsal hind flanks. These mice were supplemented with various doses of ASA (2.8, 5.6 and 11.10 mmol/L in 0.2% DMSO) in drinking water and control mice received 0.2% DMSO only. Tumor growth was monitored and assessed my measuring major and minor circumferences by digital caliper at every 3 days and tumor volume was calculated. Data are mean ± SE of 5 mice/group and expressed as tumor volume (mm^3) over the period of time (days).
